# Home-cage monitoring ascertains signatures of ictal and interictal behavior in mouse models of generalized seizures

**DOI:** 10.1371/journal.pone.0224856

**Published:** 2019-11-07

**Authors:** Miranda J. Jankovic, Paarth P. Kapadia, Vaishnav Krishnan

**Affiliations:** Department of Neurology, Baylor College of Medicine, Houston, TX, United States of America; McGill University, CANADA

## Abstract

Epilepsy is a significant contributor to worldwide disability. In epilepsy, disability can be broadly divided into two components: ictal (pertaining to the burden of unpredictable seizures and associated medical complications including death) and interictal (pertaining to more pervasive debilitating changes in cognitive and emotional behavior). In this study, we objectively and noninvasively appraise aspects of ictal and interictal behavior in mice using instrumented home-cage chambers designed to assay kinematic and appetitive behavioral measures. Through daily intraperitoneal injections of the chemoconvulsant pentylenetetrazole (PTZ) applied to C57BL/6J mice, we coordinately measure how “behavioral severity” (complex dynamic changes in movement and sheltering behavior) and convulsive severity (latency and occurrence of convulsive seizures) evolve or kindle with repeated injections. By closely studying long epochs between PTZ injections, we identify an interictal syndrome of nocturnal hypoactivity and increased sheltering behavior which remits with the cessation of seizure induction. We observe elements of this interictal behavioral syndrome in seizure-prone DBA/2J mice and in mice with a pathogenic *Scn1a* mutation (modeling Dravet syndrome). Through analyzing their responses to PTZ, we illustrate how convulsive severity and “behavioral” severity are distinct and independent aspects of the overall severity of a PTZ-induced seizure. Our results illustrate the utility of an ethologically centered automated approach to quantitatively appraise murine expressions of disability in mouse models of seizures and epilepsy. In doing so, this study highlights the very unique psychopharmacological profile of PTZ.

## Introduction

Epilepsy, or the epilepsies, are an etiologically diverse group of disorders defined by an enduring predisposition to develop epileptic seizures, and by associated neurobiologic, cognitive/psychological and social consequences [1]. Epilepsy syndromes contribute significantly to worldwide disability and premature mortality [2, 3]. In studies of adults with epilepsy, self-reported disability assessments show some correlation to seizure frequency/recency, but also correlate with anticonvulsant side effects and the intensity of comorbid mental health impairments, including anxiety and depression [4, 5]. These comorbidities magnify stigma, complicate anticonvulsant selection [6] and are associated with lower rates of seizure freedom following epilepsy surgery [7]. Understanding the etiology of these comorbidities remains an important consensus research benchmark [8] given the desperate need for safe and effective treatments that comprehensively address both seizure burden as well as interictal psychiatric derangements in patients with epilepsy.

With the appreciation of epilepsy as a spectrum disorder [9], rodent studies employing genetic and/or pharmacological manipulations popularly report seizure-related measures as well as measures of behavioral comorbidity. Video-electroencephalography remains the gold standard for the detection of spontaneous seizures, obtained via surgically implanted tethered or wireless EEG electrodes. Since spontaneous seizures in many mouse models are either rare or absent [10–12], conclusions about “enhanced seizure severity” are deduced from induced seizures. One such widely popular induction agent is pentylenetetrazole (PTZ), a gamma-aminobutyric acid type A (GABA-A) receptor antagonist [13], used briefly in the past to induce habitual seizures in epileptic patients [14]. In mice, PTZ injections produce a spectrum of behavioral changes, ranging from hypoactivity/immobility, clonic or myoclonic seizures, to tonic-clonic convulsions [15, 16]. These behavioral changes recapitulate the semiology, electroencephalographic characteristics [17] and pharmacological responsiveness [18] of seizures with a generalized onset. Differences in seizure severity are inferred from (i) subjectively scored ordinal scales of convulsive severity (e.g., Racine scale) [19–22], (ii) measures of convulsion latency or duration [17, 18], and/or (iii) rates of frank seizure-induced mortality [11]. These measures are based on the assumption that more severe seizures in mice are those that are convulsive or lethal (“convulsive severity”), arise with a shorter latency and/or are of longer duration. However, they provide no quantitative insights into how seizures interrupt/disrupt a mouse’s interaction with its environment. They also offer no information about the duration or intensity of post-ictal impairment, quantities that are critical contributors to epilepsy severity and which may also possess translational value.

Assessments of interictal emotional behavior in mice rely heavily on phenotyping batteries [23–25] to identify features of “depression-related” or “anxiety-related” behavior extrapolated from assessments of despair (e.g., forced swim test) or exploration (e.g., open field / elevated plus maze tests). While these tests are convenient and high throughput, they are short in duration (5–10 minutes long) and are often measured during the biological night (when lights are on). Virtually all of these tasks necessitate human presence/interference, which may directly impact reproducibility through inconsistent olfactory cues, variations in technique and bias [26, 27]. In this study, we employ an established home-cage monitoring platform [28, 29] to describe and appraise the acute severity of induced seizures as well as the severity of more pervasive changes in behavior that arise with recent seizures or with pervasively increased seizure risk (i.e., epilepsy). From a set of simultaneously measured elementary variables (movement, sheltering, sleep, feeding and drinking) we complement measures of convulsive severity with quantitative descriptions of the “behavioral severity” of induced seizures. Further, we extract themes of interictal behavioral dysregulation in three mouse models of generalized seizures.

## Materials and methods

### Mice and drugs

This study design was approved by the Institutional Animal Care and Use Committee (IACUC) at Baylor College of Medicine (BCM). Mice were bred and weaned at ~3 weeks of age within a vivarium set to a 12-hour light cycle (lights ON from 0500–1700) under controlled temperature (20–26°C) and humidity (40–70%) conditions. Water and chow (Pico Lab ® Select Rodent 5V5R Standard Diet) were always provided *ad libitum*. Cellulose bedding was employed (Biofresh). C57BL/6J and DBA/2J breeders (#000664 and #000671, Jackson Laboratories) obtained in October 2017 were used to generate respective inbred colonies. Scn1a R1407X mice (on a C57BL/6 background) were genotyped by polymerase chain reactions of tail DNA [30, 31] with primer sequences as follows: wildtype-forward (5’-ATGATTCCTAGGGGGATGTC-3’), mutant-forward (5’-TTTACTTTCACATTTTTCCATCA-3’), and common reverse (5’-CTTTCACATTTTTCCACCG-3’). At the end of experiments, all mice were euthanized by carbon dioxide inhalation. Among the 47 mice that were assigned to receive ten daily injections of PTZ (30mg/kg), 12 mice (4 C57BL/6J, 8 DBA2/J) died without euthanasia as a result of cardiorespiratory arrest following an induced convulsive seizure. The BCM IACUC has reviewed and approved the sudden mortality aspects of this and other protocols involving unmedicated spontaneously seizing mice. PTZ, sodium valproate (Sigma-Aldrich) and diazepam (Henry Schein) was dissolved in sterile-filtered normal saline for a final dose of 30-60mg/kg [19, 20], 200mg/kg [32] and 3mg/kg respectively [33] and injected intraperitoneally (5ml/kg).

### Home-cage monitoring

Mice were transferred into a satellite facility with lighting, light cycle, humidity and temperature settings identical to vivarium conditions. Mice were individually housed in one of sixteen Phenotyper ® (Noldus Information Technology) home-cages designed for mice (30x30x47cm) with clear plastic walls customized to incorporate two lickometered water sources (detecting capacitance changes), a feeding meter (detecting beam breaks) and a detachable running wheel (utilizing a wheel-attached magnet and a wall-attached magnet sensor). Chow and drinking water were identical to those provided in vivarium cages, and sucrose (Sigma-Aldrich) was dissolved directly into fresh drinking water at a final concentration of 0.8% (w/v). An infrared-lucent shelter (10x10x5-6cm) was also employed. A sound machine (‘Lectrofan, Adaptive Sound Technologies) played continuous white noise. Satellite access was restricted to experimenters (VK, MJJ) who wore personal protective equipment (gown, cap, face mask and gloves) and entered once daily to visually inspect food and water sources, assess general mouse wellbeing and/or administer injections. Veterinary inspections were performed every other week (~1100–1200).

### Videotracking, recordings and injections

An aerial video-feed was established using a ceiling-mounted infrared camera together with an array of ceiling mounted infrared lamps. The “shelter zone” was defined on a distance-calibrated “arena” for each cage. Live mouse center-point tracking was conducted via Ethovision XT 14 (Noldus) using dynamic subtraction at a sample rate of 15/s. Licking, feeding and wheel-running data were integrated through a hardware control module. Detection settings were consistent for all mice of any given experiment. Recordings of home cage behavior were initiated either as a “manual” start (i.e., start “now”, for ictal recordings) or started at a specific “clock” time (for baseline or interictal recordings). The “light spot” was produced by a white LED spotlight resulting in fairly diffuse cage illumination centered in the upper left quadrant of the cage. For the “cage-swap” experiment, mice were “swapped” into a cage previously inhabited by a sex-matched conspecific for a period of 2h and then swapped back into their original cages. To validate our “sleep” measurement algorithm, a separate group of C57BL/6J mice were acclimated to home-cages for 48h and then received a single injection of 3mg/kg diazepam just prior to 1700h.

### Data acquisition

For each epoch of recording (~2-3h or ~20h in duration), data pertaining to horizontal distances was automatically tallied by Ethovision XT 14 and subsequently smoothed to ignore horizontal displacements of less than 0.2cm (between samples): this was necessary to cancel out oscillatory tracking noise in mice that were either extremely immobile or deceased. Distance, licking duration, feeding duration (or entries) and sheltering time were reported as daily totals (for time budgets) or binned as either total/minute or total/hour. Convulsive seizures were defined broadly as any abrupt paroxysm that resulted in a loss of upright posture, clonic and/or tonic movements, with or without wild running, and/or hind limb extension (S3 and S4 Movies). Convulsive events were identified by screening “ictal recordings” through integrated visualization (S2 Movie), where changes in “% mobility” (defined as the percentage of *changed pixels* of an object between samples) were reviewed simultaneously with video data. For each “ictal” recording, all spikes in % mobility were screened and the latencies of true convulsive seizures (confirmed by video review) were reported.

### Data analysis

Graphs and statistical analyses were generated on Prism GraphPad 8. To assess total sleep time and sleep bout duration, a stand-alone module was designed to analyze Ethovision raw data files and identify 40s-long contiguous periods devoid of “movement” (defined as sample velocity ≤1.2cm/s). When mice died following a PTZ injection, death was tallied in survival curves and convulsion occurrence and latency were included in the analysis. However, data from that specific PTZ injection was excluded from any graphs of averaged “ictal” behavior. These deaths also resulted in missing values for daily total measurements. To perform repeated measures analysis of variance (RMANOVA) with missing values, a mixed effects model was employed, and interactions between fixed effects are reported (e.g., *group x time*, or *group x day*). Unpaired two-tailed Student’s *t* tests were used to compare two group means. Identical p values were obtained using Welch’s *t* test, which does not assume equal variances. Two-tailed Chi squared analyses were employed to demonstrate significant changes in the *fractions* of mice that convulsed.

## Results

### Spontaneous home-cage behavior in wildtype C57BL6/J mice

**Fig 1:**
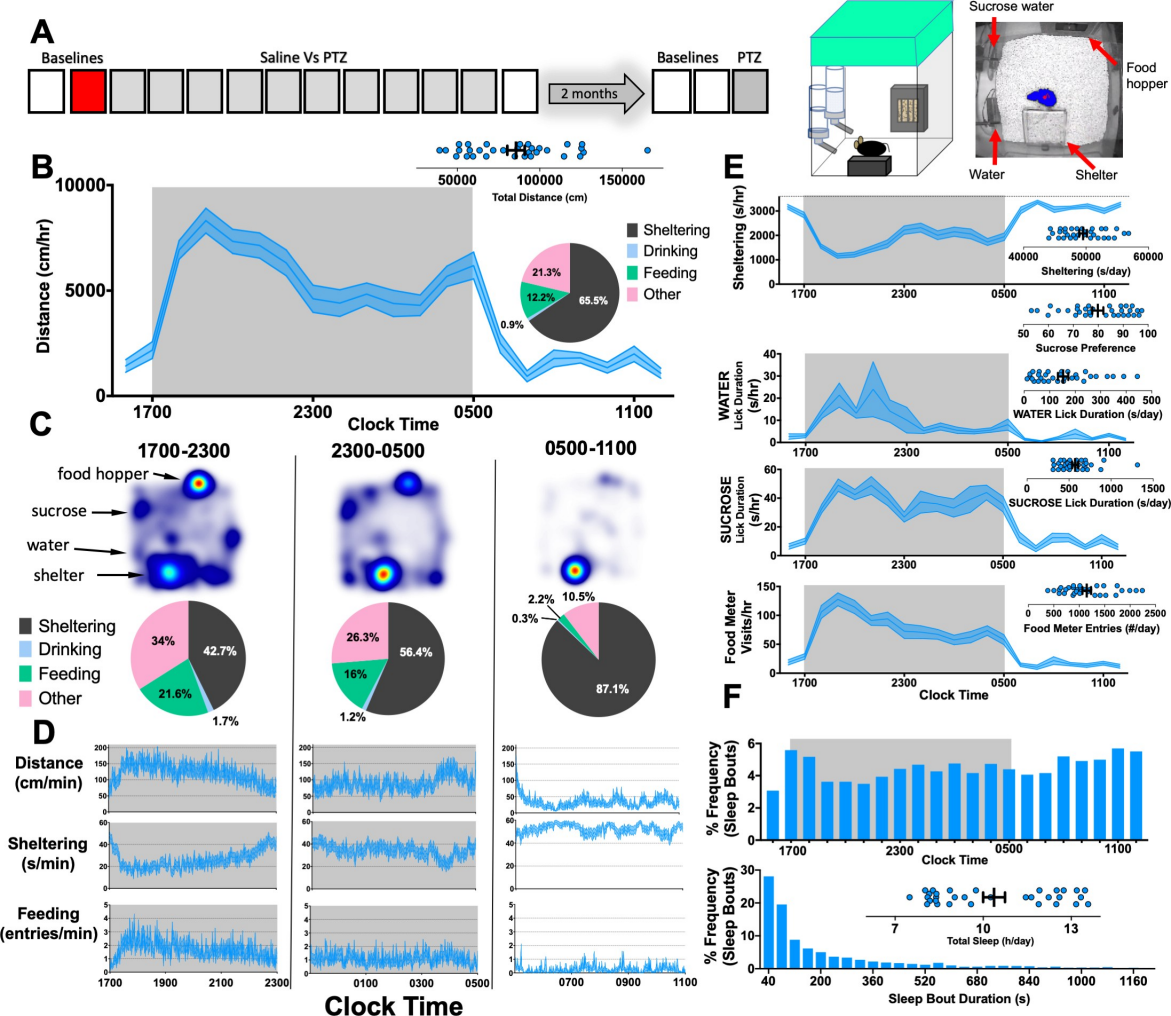
Home-cage behavior in C57BL/6J mice. **(A)** Experimental protocol for 8-week old C57BL/6J mice (n = 32, 16 female), with the epoch depicted by this figure in marked red. RIGHT: Cartoon showing home-cage configuration with a screen capture from an aerial infrared camera showing mouse body contour (blue) and centerpoint (red). **(B)** Distances moved (per hour) on baseline day 2, with shaded box depicting the active phase. INSET: time budget across this 21h recording period. **(C-D)** Heat maps, time budgets and behavioral quantities depicted over 6h long epochs. “Other” is defined as time spent *not* sheltering, drinking or feeding. **(E)** Average rates of sheltering, licking and feeding measured simultaneously with individual total values plotted in inset. **(F)** Percent frequency of sleep bouts as a function of time of day (TOP) and by duration of sleep bout (BOTTOM), with individual values obtained for total sleep (INSET). Mean ± standard of the mean (SEM) shown.

Adult C57BL/6J mice were individually housed in home-cage chambers containing an infrared—translucent shelter, two lickometered water sources (water Vs 0.8% sucrose) and a beam-break metered food hopper. Before intraperitoneal injections, we obtained two days of baseline recordings (lights are off between 1700–0500, defined as the *“*active phase”). Here, we profile data from the second day (Fig 1A). Between 1500–1200, mice accumulated an average total distance of ~850m (Fig 1B). Heat maps of position probability during 6h-long epochs demonstrated early peaks at the food hopper (1700–2300) and late peaks in the shelter (2300–1100). Averaged time budgets [34] quantified this effect (Fig 1C): these were calculated by summating the total durations (per epoch of time) spent at the feeder, water spouts, shelter or engaged in “other” behaviors (time spent *not* feeding, drinking or sheltering). Consumptive and sheltering behavior were generally synchronized with locomotor activity (Fig 1D and 1E), and mice displayed an average sucrose preference of ~80%. To measure “sleep” noninvasively, we identified contiguous periods of lack of movement that were ≥ 40s in duration (see S1A Fig for a pharmacological validation of this technique with diazepam). This approach displays ~90% agreement with sleep states derived from electroencephalography-electromyography but does not discriminate rapid eye movement (REM) sleep from non-REM sleep [35, 36]. C57BL/6J mice on average spent ~10.3h in “sleep”, with sleep bouts (mean duration ~263s, Fig 1F) occurring throughout the day consistent with prior reports [37–39]. Together, these data recapitulate (in a laboratory setting) natural circadian patterns of foraging and sheltering behaviors of mice in the wild, achieved entirely through automation and without concurrent human presence or surgical implants [40].

### Acute effects of intraperitoneal pentylenetetrazole injections

**Fig 2:**
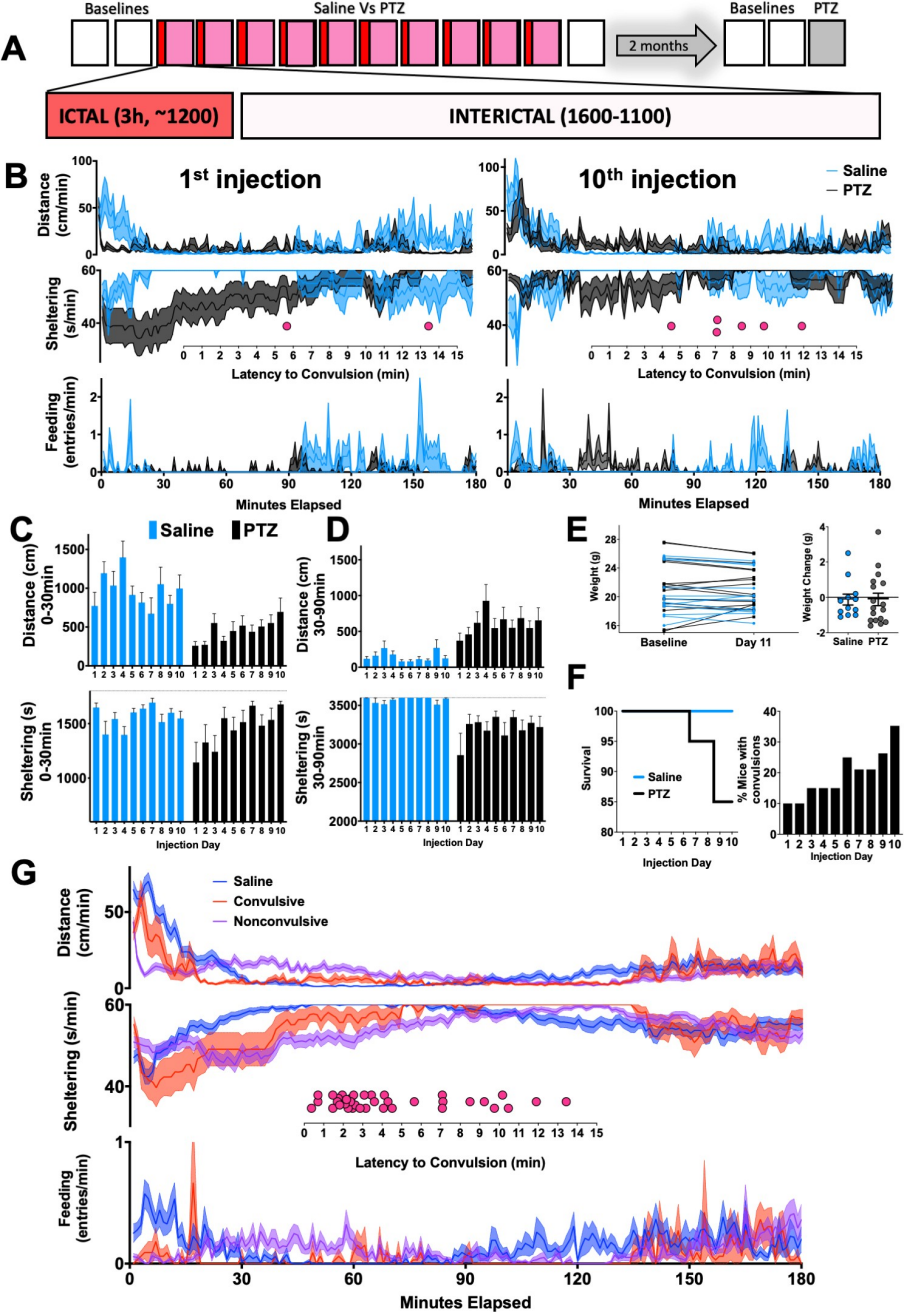
Acute changes in home-cage behavior. **(A)** 10 daily 3h-long “ictal” recordings began at approximately noon, following injections of either saline (n = 12) or 30mg/kg PTZ (pentylenetetrazole, n = 20) **(B)** Changes in distance moved, sheltering and feeding observed during the 1^st^ and 10^th^ injections (see also S2 Fig). Dot plots depict latencies to convulsions. **(C)** In the first 30 minutes, total distance and sheltering time increased over the 10-day injection period (distance: group x day, F_9,261_ = 2.95, p<0.01, and sheltering: group x day, F_9,261_ = 2.00, p<0.05). **(D)** In the subsequent hour (30-90mins), total distances (group x day, F_9,261_ = 0.91, p>0.05) and sheltering (group x day, F_9,261_ = 0.95, p>0.05) did not significantly evolve. **(E)** Weight change between groups (p>0.5). **(F)** Survival and occurrence of convulsions over the 10-day protocol. **(G)** Ethograms averaged across all 10 days for saline injections (n = 120), convulsive (n = 33) and nonconvulsive (n = 158) ictal recordings (inset: latency to convulsion across all days). Mean ± SEM shown.

We randomized these mice into two sex-matched groups (S1B–S1E Fig) to receive 10 daily intraperitoneal injections of either saline or PTZ. We chose a dose of 30mg/kg, generally considered to be “subconvulsant”, and therefore an ideal dose to induce chemical kindling [16, 19, 20, 41]. Injections were administered at ~1200 for three reasons: (i) to respect how many epilepsy mouse models display peaks of spontaneous seizure occurrence during the inactive phase [42–44], (ii) to avoid light-induced circadian disruptions that invariably occur when injections are administered with lights off, and (iii) to elaborately emphasize how daily seizures impact the entirety of active phase behavior. Immediately following injections, we closely analyzed 3h-long “ictal” recordings as well as more prolonged “interictal” recordings (1600 to 1100, Fig 2A). On the first injection day (Fig 2B), saline-treated mice displayed a gradual reduction in locomotor activity over ~30 minutes, following which they retreated to a state of marked immobility (30–90 minutes) within shelters. Approximately 90 minutes following saline injections, mice resumed normal rates of daytime movement and sheltering behavior (compare with Fig 1D). Over subsequent days, behavioral responses to saline injections remained fairly stereotyped (Figs 2B–2D and S2).

In contrast, the first PTZ injection produced an early steep decline in locomotion associated with a significant sheltering deficit (Fig 2B, S1 Movie). With repeated PTZ injections, this early sheltering deficit and immobility response gradually improved (*group distance x day*, p<0.01, *group sheltering x day*, p<0.05, Fig 2C). Between 30–90 minutes after the first injection, PTZ-treated mice displayed an unexpected relative increase in locomotion with continued deficits in sheltering (Fig 2B). Relative hyperactivity and sheltering deficits (during this epoch) did *not* substantially evolve with repeated PTZ injections (Fig 2D). Weight change, assessed over the 10 day protocol, was no different between these groups (Fig 2E).

Through an integrated visualization of video and real-time measures of mobility (see Materials and Methods, S2 Movie), we retrospectively identified the occurrence of convulsive seizures (37 in total), which as expected, increased in probability with repeated PTZ exposure (Fig 2F, S2 Fig, S3 and S4 Movies). Convulsions occurred with a mean latency of 4.44 min, and a total of three convulsions resulted in sudden death. Mice that displayed any convulsions during the entire injection period (9 out of 20) trended to be more hyperactive (at baseline) than those that never convulsed (*group x hour*, p = 0.1, S3A Fig). 7 out of 20 mice displayed convulsions following the second or subsequent PTZ injections. When compared with the 11 mice that never convulsed, these mice displayed significantly lower amounts of sheltering between 30–90 minutes after their *first* injection of PTZ (p<0.05, S3B Fig). Across all 10 injection days (S3C Fig), PTZ injections on average resulted in a marked but transient sheltering deficit (0–90 min) together with early relative hypoactivity (0–30 min) and late relative hyperactivity (30–90 min). When this average response was separated by the presence or absence of a convulsion (Fig 2G), distinct patterns of behavioral severity were revealed: convulsive seizures resulted in an early activity “spike” followed by a protracted period of behavioral quiescence (similar to saline-treated mice). In contrast, nonconvulsive seizures were associated with a more sustained sheltering deficit together with relatively increased exploratory activity within 30–90 minutes after the injection.

### The interictal behavioral syndrome induced by PTZ injections

**Fig 3:**
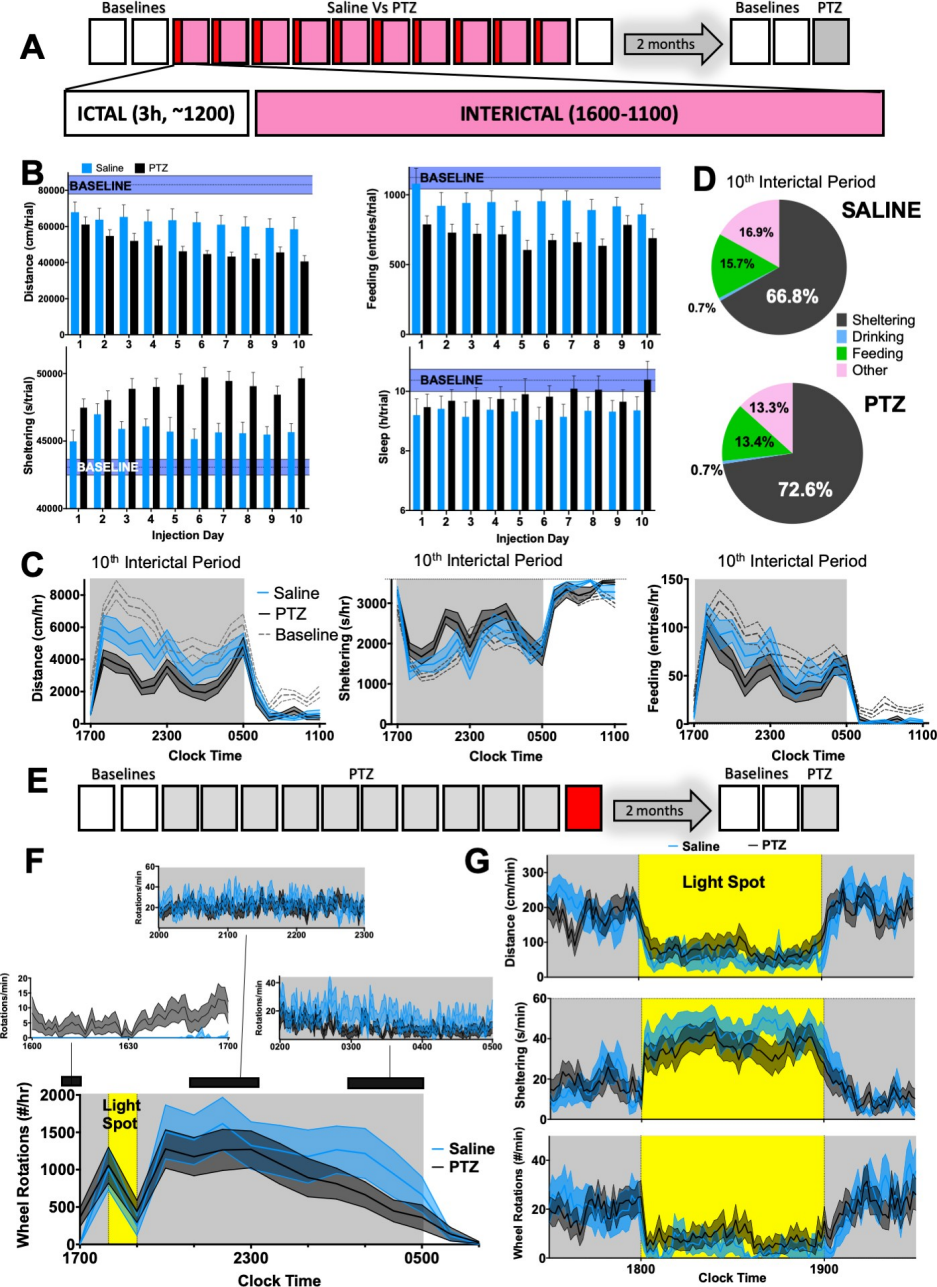
Daily PTZ injections produce an interictal syndrome of hypoactivity and increased sheltering. **(A)** 10 daily 16h-long “interictal” recordings beginning at 1600 (saline n = 12, PTZ n = 17–20) **(B)** PTZ injections produced hypoactivity (group x day, F_9,261_ = 1.64, p = 0.1), increased sheltering (group x day, F_9,261_ = 2.17, p<0.05), without changing feeding (group x day, F_9,261_ = 0.63, p>0.5) or sleep (group x day, F_9,261_ = 0.66, p>0.5). Baseline ranges (corresponding to total measurements between 1600–1100 for day 2) are depicted in purple (mean + SEM) **(C)** 10^th^ interictal period: PTZ resulted in hypoactivity (group x hour, F_18,486_ = 2.2, p<0.01), a trend for increased sheltering (group x hour, F_18,513_ = 1.47, p = 0.09) without altering feeding (group x hour, F_18,486_ = 0.97, p = 0.4). **(D)** Averaged time budgets for C. **(E)** On the day after their last PTZ or saline injection, **(F)** voluntary wheel running was measured. PTZ-treated mice displayed increased wheel running during the first hour (saline 0.1±0.05 Vs PTZ 6.4±2 rotations/min, p<0.05), but similar wheel running during the mid-active phase (2000–2300, saline 24.1±5.3 Vs PTZ 20.6±4.2 rotations/min, p>0.1) or during the late active phase (0200–0500, saline 15.7±4.7 Vs PTZ 8.1±1.8 rotations/min, p>0.5). **(G)** A light spot test produced similar changes in activity and sheltering in PTZ and saline-treated mice. Mean ± SEM shown.

We obtained 19h long “interictal” recordings of spontaneous home-cage behavior after every injection to quantify more delayed behavioral differences (Fig 3A). The experience of saline injections alone reduced total daily distances to ~600m/d, but this remained fairly stable with repeated injections. In comparison, PTZ-treated mice displayed a progressive relative decline in total locomotor activity and increased sheltering (*group distance x day*, p = 0.1, *group sheltering x day*, p<0.05, Fig 3B) but without increases in overall “sleep”, sucrose preference or total licking (S4A Fig) On many but not all injection days, PTZ-treated mice displayed fewer entries into the food meter (Fig 3B). Sucrose preference and total licking was similar in PTZ and saline groups (S4A Fig). The 10^th^ interictal period is profiled in Fig 3C, where hypoactivity and increased sheltering behavior were most prominent during the active phase (*group distance x day*, p<0.01, *group sheltering x day*, p = 0.09). PTZ-treated mice spent more time sheltering at the “cost” of reduced feeding and “other” behaviors (Fig 3D), reflecting a more general reduction in home-cage exploration. Across all interictal recordings, convulsive seizures were associated with more prominent increases in sheltering and hypoactivity (S4B Fig).

We incorporated two additional perturbations the day following their last injection to explore associated changes in hedonic drive and risk aversion (Fig 3E). First, we introduced running wheels just before 1600. During the first hour of wheel exposure, PTZ-treated mice accumulated significantly more wheel rotations (p<0.05, Fig 3F). Overall rates of nocturnal wheel-running were largely similar between groups, including at active phase onset, when both groups mounted a robust increase in wheel running (~1000 rotations/hr or ~471m/hr, Fig 3F). Second, at 1800, we applied a 60-minute long “light spot” stimulus [45], which interjects a conflict between nocturnal foraging behavior and light aversion. This light stimulus attenuated locomotion and enhanced shelter entry, but saline and PTZ-treated mice displayed largely similar responses (Fig 3G).

### Delayed effects on seizure severity and anticonvulsant pre-treatment

**Fig 4:**
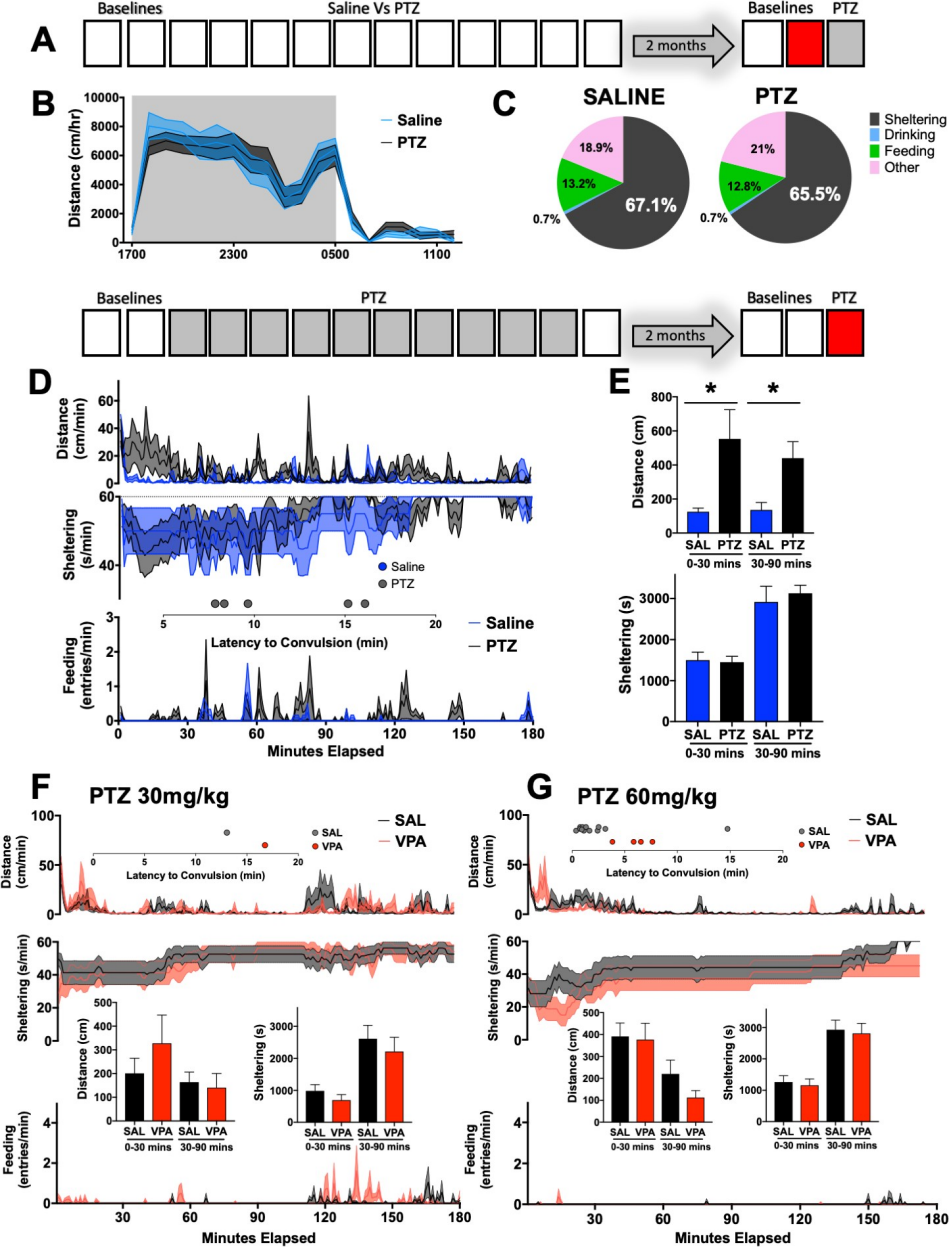
Home-cage behavior after seizure remission and anticonvulsant treatment. Two months after the cessation of daily injections, mice were reintroduced into home-cage monitoring chambers. **(B)** Overall distances moved between previously saline (n = 12) and PTZ-treated (n = 17) mice were no different (group x hour, F_19,513_ = 0.44, p>0.5. **(C)** Time budgets for epoch shown in B. **(D)** Final injection of PTZ (30mg/kg): previously saline-treated mice displayed marked immobility, whereas previously PTZ-treated mice displayed relative hyperactivity together with convulsions (PTZ 5/17 vs Saline 0/12, χ^2^ = 4.2, p<0.05). **(E)** Quantification of findings in D. Distances, sheltering and feeding responses following a **(F)** 30mg/kg or **(G)** 60mg/kg PTZ injection approximately one hour after an intraperitoneal injection of saline or 200mg/kg VPA (sodium valproate, n = 16, 8 females/group). Valproate-treated mice displayed significantly fewer convulsions in response to 60mg/kg PTZ (4/16 Vs 13/16, χ^2^ = 10.1, p<0.01). * p<0.05. Mean ± SEM is shown.

The interictal syndrome identified in the midst of repeated PTZ injections was absent when the same mice were studied two months later (Fig 4A–4C, S4C Fig). After collecting baseline recordings at this late time point, all mice received a final injection of PTZ (30mg/kg). Previously saline-treated mice displayed pronounced immobility (p<0.05). In contrast, previously PTZ-treated mice displayed a significantly greater number of convulsions (p<0.05) together with early relative hyperactivity (p<0.05, Fig 4D and 4E), similar to the “kindled” response seen following the 10^th^ PTZ injection in Fig 2B.

Next, in order to examine how anticonvulsant pre-treatment would impact behavioral and/or convulsive measures of seizure severity, a separate group of 8-10week old mice were acclimated to home-cages for 48h. Rather than screen multiple anticonvulsants, we selected valproic acid as an anticonvulsant known to be effective in the PTZ model [18, 32] and generally efficacious in the management of idiopathic or symptomatic generalized epilepsies [46]. Mice received either an injection of saline or sodium valproate [200mg/kg] [18, 32], followed 1h later by a 30mg/kg PTZ injection. Valproate pre-treatment did not significantly affect behavioral severity or the occurrence of convulsions with a subconvulsant PTZ dose (Fig 4F). The following day, we applied similar saline or valproate injections an hour before a convulsant dose of PTZ (60mg/kg) [20]. As expected, valproate resulted in significantly fewer convulsions. However, dynamic measures of distance and sheltering behavior were similar between groups.

### Comparing C57Bl/6J and DBA/2J mice

**Fig 5:**
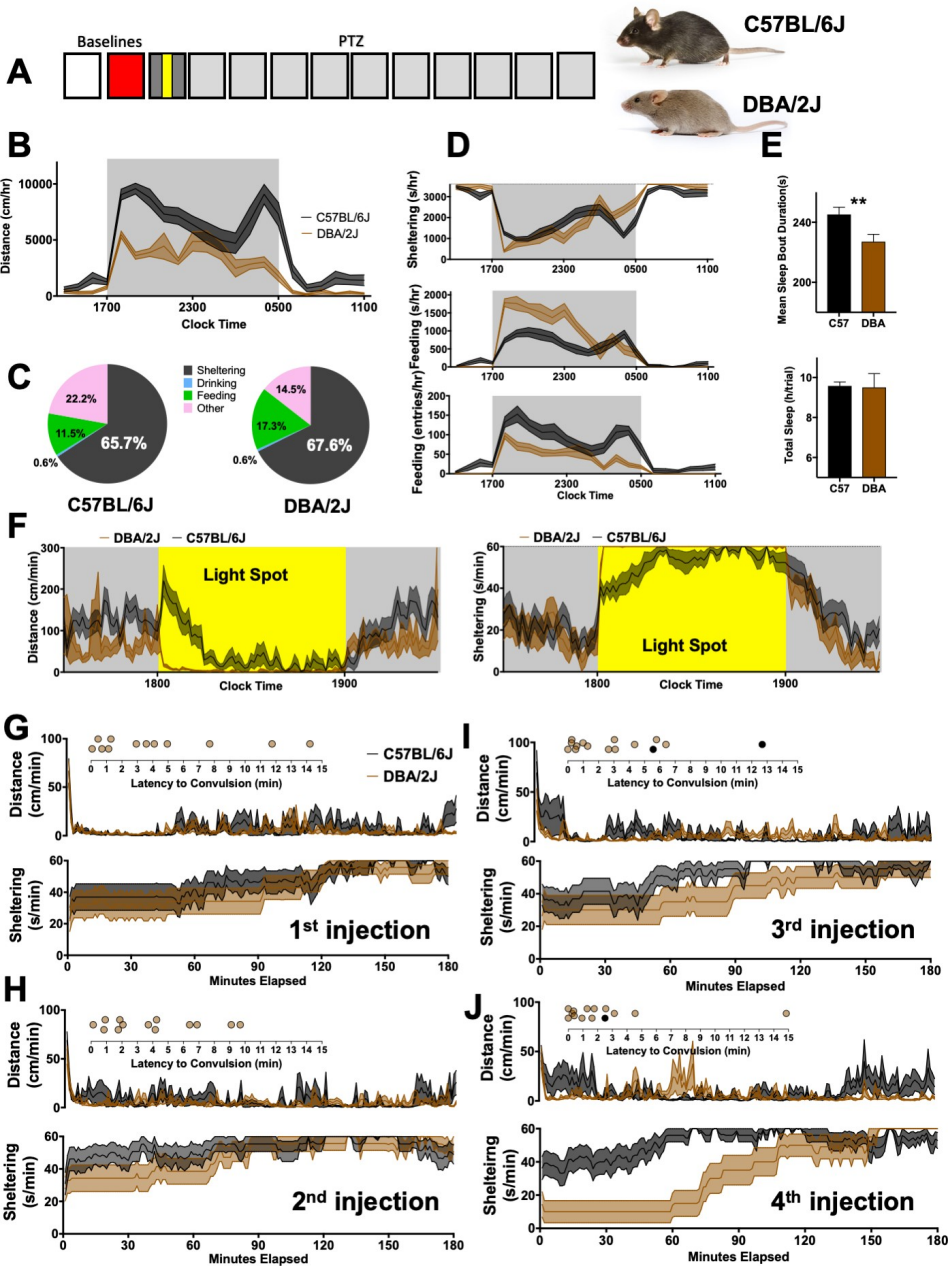
C57BL/6J Vs DBA/2J. **(A)** 8-10week old C57BL/6J (n = 15, 7 female) and DBA/2J (n = 15, 7 female) were directly compared. Photographs with permission from © The Jackson Laboratory. **(B)** On the second baseline day, DBA/2J mice were markedly hypoactive (genotype x time, F_21,588_ = 5.9, p<0.0001). Overall time budgets are shown in **(C)**. **(D)** DBA/2J mice also displayed significantly different patterns of sheltering (genotype x hour, F_21,588_ = 4.3, p<0.0001), increased feeding duration (genotype x hour, F_21,588_ = 8.7, p<0.0001) and decreased feeding entries (genotype x hour, F_21,588_ = 3.8, p<0.0001). **(E)** Mean sleep bout duration and total sleep time. **(F)** During the light spot hour, DBA/2J mice displayed rapid and committed shelter entry and immobility. **(G-I)** Distances and sheltering patterns for the first-through fourth daily injections of PTZ in ~10week old female DBA/2J (n = 14) and C57BL/6J (n = 13) mice with insets depicting the occurrence of convulsive seizures (see also S5 Fig). ** p<0.01. Mean ± SEM is shown.

Compared with C57BL/6J mice, mice of the DBA/2J strain display a greater incidence and a diminished latency to convulsive seizures following exposure to PTZ and the related GABAergic antagonist fluorothyl [15, 47]. To explore how such differences in seizure threshold may associate with changes in home-cage behavior, we compared age and sex-matched C57BL/6J and DBA/2J mice bred under identical conditions (p<0.0001, Fig 5A). In addition to hypoactivity, DBA/2J mice also displayed lower entries but a greater duration of time at the food hopper (p<0.0001, Fig 5B–5D). DBA/2J mice had shorter mean durations of “sleep” bouts without significant differences in overall “sleep” (Fig 5E). In response to a light spot (Fig 5F), DBA/2J mice displayed rapid and sustained shelter entry together with prominent locomotor suppression for the duration of the light stimulus. Importantly, at this age, both strains are similar in light detection and visual acuity [48].

Next, we asked whether changes in PTZ-induced convulsive severity in DBA/2J were associated with similar changes in behavioral severity. In preliminary experiments, a high rate of PTZ-induced mortality was seen in male DBA/2J mice: in a cohort of 8 male DBA/2J mice, only 2 survived past the sixth PTZ injection. Therefore, here we present data from only female mice (of both inbred strains). Following the first PTZ injection, C57BL/6J and DBA/2J mice displayed similar patterns of locomotor activity and sheltering behavior, but DBA/2J displayed a higher incidence of convulsions (Fig 5G). Over subsequent days of testing, DBA/2J continued to display the vast majority of convulsions and experienced seizure-induced mortality at a greater rate (Fig 5H–5J, S5 Fig). In several but not all of their subsequent PTZ injections, DBA/2J mice exhibited marked delays to shelter re-entry (S5 Fig).

### Ictal and interictal measures in a mouse model of Dravet syndrome

**Fig 6:**
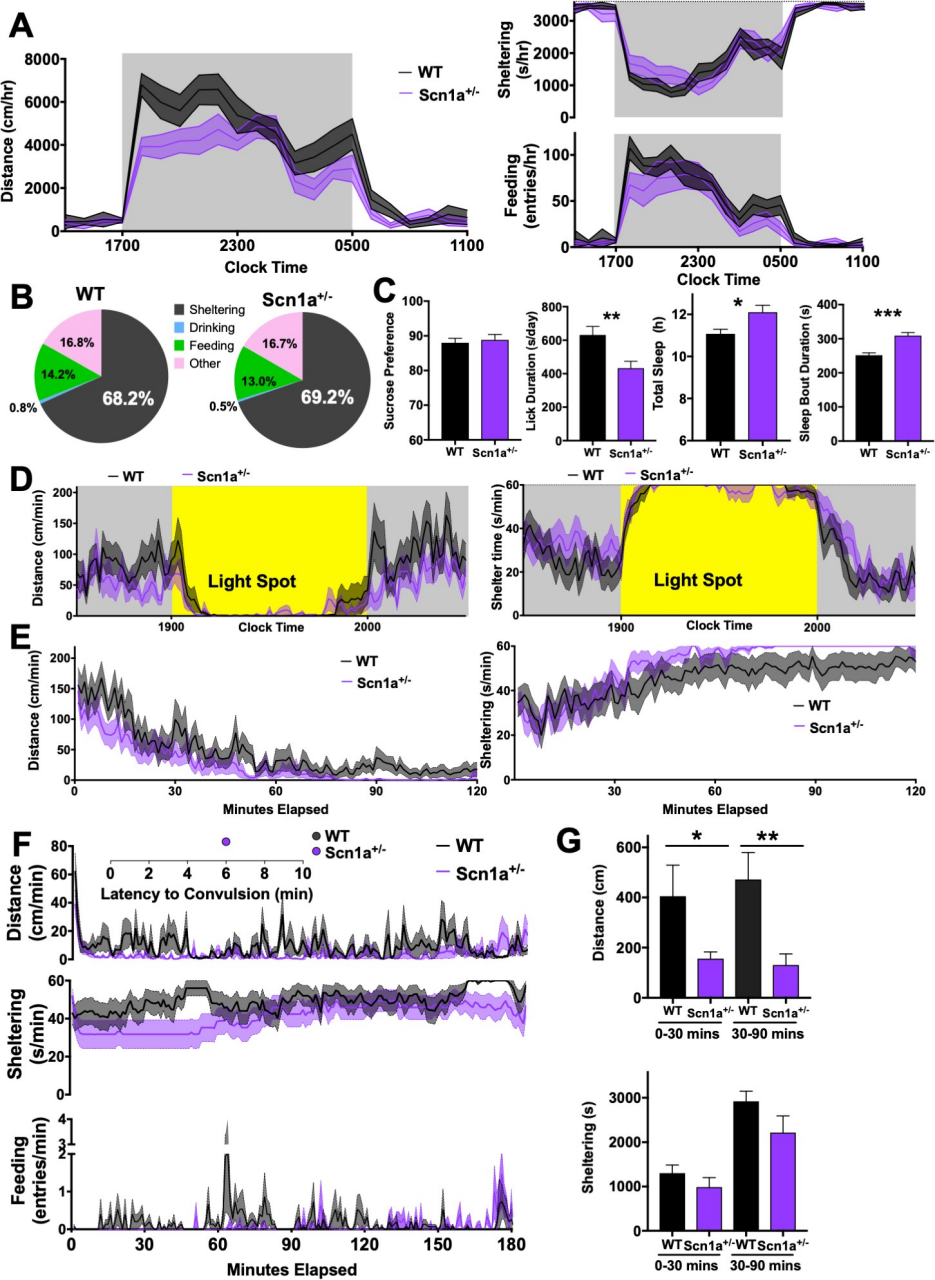
Home-cage behavior in Scn1a^+/-^ mice. **(A)** 8-10week-old Scn1a^+/-^ mice (n = 15, 8 male) were hypoactive compared with littermate WT mice (n = 13, 8 male, genotype x hour F_21,546_ = 2.21, p<0.01**)** without significant differences in sheltering or feeding entries (p>0.1). **(B)** Time budgets for A. **(C)** Scn1a^+/-^ mice displayed lower overall total licking without a change in sucrose preference, together with increased total sleep time and mean sleep bout durations. **(D)** Light spot stimulation revealed no differences, but **(E)** with a day time “cage-swap”, Scn1a^+/-^ mice displayed more rapid locomotor habituation and accelerated shelter entry. **(F)** Following PTZ (30m/kg), Scn1a^+/-^ mice displayed greater immobility, quantified in **(G).** WT = wildtype. * p <0.05, ** p<0.01, *** p<0.001. Mean ± SEM is shown.

Dravet syndrome (DS) is a neurodevelopmental disorder characterized by intellectual disability, autism spectrum disorder and epilepsy [49]. Mutations in the voltage gated sodium channel subunit *SCN1A* are found in approximately 80% of patients with DS [50] and mice engineered to heterozygously express a clinically observed truncation mutation in *Scn1a* (R1407X, hereafter referred to as Scn1a^+/-^) display rare spontaneous seizures with a normal interictal electroencephalogram [30, 31, 51]. Adult Scn1a^+/-^ mice have been reported to display altered anxiety-related behaviors, impaired sociability and spatial memory [52]. In our home-cage chambers, compared with wildtype littermates, Scn1a^+/-^ mice displayed early active phase hypoactivity (*group x distance*, p<0.01), without alterations in feeding and sheltering (Fig 6A). Like PTZ-treated mice (Fig 3D), Scn1a^+/-^ mice spent more time sheltering at the cost of reduced feeding (Fig 6B). Overall licking was reduced (p<0.01) without affecting sucrose preference, and Scn1a^+/-^ mice displayed significantly higher total “sleep” (p<0.05) and longer bouts of “sleep” (p<0.001, Fig 6C). In response to a light spot stimulus [45], Scn1a^+/-^ mice and WT littermates performed similarly (Fig 6D). In contrast, Scn1a^+/-^ mice displayed hypoactivity and accelerated shelter entry following a temporary (2h long) “cage swap” (Fig 6E). Finally, in response to 30mg/kg of PTZ, Scn1a^+/-^ mice displayed prominent immobility during both early (0-30min, p<0.05) and late (30-90min, p<0.01) epochs (Fig 6F and 6G). Scn1a^+/-^ mice also displayed a prominent suppression of feeding over the entirety of the 3h long observation period. Only one Scn1a^+/-^ mouse convulsed.

## Discussion

Home-cage monitoring technology has unearthed important behavioral phenotypes in mouse models of autism [53–55], neuromuscular and movement disorders [56, 57] and Alzheimer-related cognitive impairment [58, 59]. Since the behavioral expression of a static epileptogenic lesion may depend upon the timing of the most recent seizure, we submit that home-cage behavioral monitoring (especially when combined with EEG) is particularly well suited to study the dynamics of interictal behavior in mouse models of epilepsy. Measuring multiple variables simultaneously over prolonged recording periods avoids biased assumptions about *when* (i.e., the time of day) or *how* (i.e., which behavioral axis) a genetic or pharmacological manipulation may be symptomatic [26]. Such home-cage assessments may provide superior translational value, especially when aligned with human data captured by wearable devices designed to quantify sleep, sedentariness and frailty.

### Distinguishing behavioral severity and convulsive severity

The first objective was to apply home-cage monitoring to more comprehensively and objectively measure “seizure severity”, an endophenotype central to rodent studies designed to identify and demonstrate pharmacological or genetic modulators of epilepsy or epilepsy risk. In experiments with PTZ, seizure severity has often been equated with convulsive severity: *ordinal* scales designed to quantify seizure severity assign low scores to hypoactivity/immobility (assessed subjectively), intermediate scores to myoclonic seizures (which are often subtle), and high scores to convulsive or maximal seizures, often with even higher scores for seizures associated with death [15, 16, 19, 22]. Our data showed that in the first 30 minutes, PTZ-treated mice (compared with saline) displayed profound immobility and a significant sheltering deficit. In experiments with simultaneous EEG, PTZ injections in mice have been shown to induce bursts of repetitive (6-7Hz) and/or isolated generalized spike-wave discharges for approximately 15–20 minutes [17, 18]. Thus, we hypothesize that PTZ-induced immobility *outside* the shelter may represent a transient epileptic encephalopathy, akin to “spike-wave stupor” [60]. From an ethological perspective, since immobility *outside* the shelter during the day time (S1 Movie) would render a mouse more susceptible to predation, the extent of immobility outside the shelter must be proportional to severity. Interestingly, with subsequent PTZ injections, mice displayed a progressive *improvement* in these early measures (Fig 2C), albeit at the cost of a progressive increase in the likelihood of convulsion. Convulsive seizures always occurred within the 30 minutes after PTZ injections and were identified manually: since individual “spikes” in horizontal displacement or mobility were not always related to convulsions, and since not all convulsions necessarily resulted in such “spikes”, we utilized a trained blinded observer to score the presence and timing of convulsive seizures.

In the subsequent hour (between 30–90 minutes post-injection), saline-treated mice displayed profound hypoactivity (~1-2cm/min) and a committed prolonged shelter entry. In contrast, PTZ-treated mice continued to ambulate (~20-30cm/min), intermittently exited their shelters and entered their food hopper, resembling a “wandering” state [61]. This response would *also* render mice vulnerable to predation, and therefore must *also* relate to severity. We observed that repeated PTZ injections did not significantly change these putative measures of wandering (Fig 2D). Thus, while “convulsive severity” may display a simple linear or step wise worsening daily injections of PTZ (Fig 2F) [16, 17, 19, 20], changes in behavioral severityare more complex. Indeed, both early and late/kindled PTZ-induced seizures were severe in unique ways (Fig 4D). Similarly, convulsive and nonconvulsive seizures distinctly perturbed home-cage behavior (Fig 2G), and aside from the convulsion itself, we found no quantitative behavioral evidence to suggest that convulsive seizures are necessarily more “severe”. Distinctions between convulsive and behavioral severity were evident with our other manipulations. Two months following daily injections, PTZ re-exposure resulted in a greater incidence of convulsions in previously PTZ-treated mice, whereas previously saline-treated mice displayed significant immobility without any convulsions. Valproate pretreatment improved convulsive severity without impacting behavioral severity (Fig 4G); this may be related to the inability of valproic acid to completely eliminate PTZ-induced spike-wave discharges [18]. DBA/2J mice (compared with C57BL/6J) displayed greater convulsive severity (Fig 5G) without altering the early behavioral responses to PTZ. In contrast, Scn1a^+/-^ mice (compared with wild type littermates) displayed greater behavioral severity in response to subconvulsant dose PTZ without a significant increase in convulsion occurrence (Fig 6F). Together, these results illustrate how objective home-cage derived measures of PTZ-induced behavior identify aspects of seizure severity that may not be captured by ordinal scales.

### The severity of interictal behavioral dysregulation

The second objective of the study was to explore how (i) seizure recency (or frequency) and/or (ii) an increased seizure susceptibility (i.e., epilepsy) impacts spontaneous home-cage behavior. During the 10-day injection protocol, PTZ-treated mice gradually developed a syndrome of hypoactivity (without excess sleep) and increased overall sheltering behavior. On average, these responses were more pronounced following those PTZ injections which resulted in convulsions (S4B Fig), consistent with the widely observed phenomenon that generalized or secondarily generalized convulsions result in a more lasting and significant post ictal impairment. There were no associated deficits in hedonic drive (sucrose preference, running wheel) or exploratory behavior (light spot, running wheel). Further, this syndrome was transient: it was not evident two months after the cessation of daily PTZ injections. Nevertheless, at this distant time point, persistent differences in “seizure severity” could be demonstrated with a subsequent injection of PTZ. These findings illustrate how interictal behavioral changes that emerge *during* a period of frequent seizures may remit with seizure remission, *without* altering the threshold or the severity of a subsequent provoked seizure.

Prior to PTZ exposure, DBA/2J mice displayed prominent home-cage hypoactivity compared with C57BL/6J, replicating findings from other home-cage platforms designed to measure activity or wheel-running only [27, 28, 62]. In our analysis, hypoactivity could not be explained by excess “sleep” but was associated with (or a result of) fewer but more prolonged feeding bouts. In response to light spot stimulation, DBA/2J mice were markedly different from C57BL/6J mice, with rapid shelter entry and locomotor suppression. While increased sheltering during the light spot test has been advertised as “anxiety-like” [45, 63], mice in the wild naturally *decrease* exploratory behavior under conditions of illumination (e.g., moonlit nights) as an innate response to increased predation risk [40]. Thus, enhanced sheltering in response to light spot testing may be a more general measure of *risk aversion*, which may itself be modulated by other factors such as cognitive dysfunction, sympathetic activation and satiety/thirst. Scn1a^+/-^ mice also displayed nocturnal hypoactivity together with a subtle but statistically significant increase in total “sleep”. With light spot testing (conducted without concurrent human presence), suppression of exploration and shelter entry were largely similar between Scn1a^+/-^ mice and littermate controls. In contrast, when mice were transferred (by a human experimenter) into an adjacent cage with novel olfactory cues (“cage swap”), Scn1a^+/-^ mice displayed lower exploratory activity and swifter shelter entry. These data exemplify how differences in exploratory tendency may certainly be dependent on the nature of the test, timing and the presence of a human experimenter.

Nocturnal home-cage hypoactivity that we observed in all three models (repeated PTZ, DBA/2J and Scn1a^+/-^ mice) is itself etiologically nonspecific. Similar observations have been made in some [53–55] but not all [64, 65] mouse autism models, following social defeat stress [66] and also in aged mice [67]. Indeed, wide variations in spontaneous home-cage activity and wheel-running exist amongst genetically diverse inbred strains of mice [28, 62]. Hypoactivity may either promote survival (by conserving energy and limiting predation) or compromise survival (by lowering access to food/water and mating). Drowsiness and/or a post-ictal encephalopathy could also conceivably result in hypoactivity. Light spot testing did *not* reveal a blunted or delayed response in either of our three models. Thus, at least during the early active phase (when the light spot was presented), observed hypoactivity was not associated with evidence for an impaired “sensorium” (at least to an aversive visual stimulus).

### Limitations

First and foremost, our technique does not (currently) incorporate simultaneous electroencephalography (EEG). Thus, our approach fails to provide a temporally precise appreciation of how the presence or absence of intermittent epileptiform discharges may correlate with putative “stuporous” or “wandering” behavior. In the same light, we are unable to discern how the timing of spontaneous seizures in adult Scn1a^+/-^ mice (albeit rare [52]) may transiently alter home-cage behavior. Combining long term home-cage monitoring with synchronized high-quality wireless EEG remains an important ongoing objective but may reveal *observer effects* across both ictal and interictal measures: the mere placement of EEG electrodes in mice (tethered *or* wireless) may impair mobility, sleep and neurovegetative function through pain or direct physical hindrance. Further, EEG implantation surgery may itself increase seizure threshold through pain, impaired sleep and postoperative inflammation [68], as well as through unintended cortical microlesions that may occur when electrodes are inadvertently advanced to subdural depths [69].

Second, this report describes seizures artificially induced by a single chemoconvulsant, PTZ. We chose this approach, as it allowed us to induce seizures “on-demand” in large cohorts of mice, thereby synchronizing our assessment of ictal and interictal behavior. It also enabled us to model a brief period of frequent seizures followed by remission. While acute seizure protocols are invaluable to screen and identify novel anticonvulsants [12], their relevance to infer the pathophysiology of epilepsy (or epilepsy comorbidities) is admittedly inferior to models that display frank spontaneous seizures. Since PTZ is a GABA-A receptor antagonist [13], differences in induced convulsive or behavioral severity may be a reflection of primary alterations in GABA-A receptor expression and/or subunit composition. Such molecular differences may manifest as apparent alterations in seizure severity (i) across different inbred strains of mice, (ii) between wild type and mutant littermates (e.g., Scn1a^+/-^ mice), or (iii) within individual mice of a particular inbred strain.

Separately, the effects of PTZ on interictal behavior may be related to the overall severity of the induced seizure or the pharmacodynamic impacts of daily transient GABA-A receptor antagonism. PTZ at lower doses is employed as a model of induced transient anxiety and has been shown to ameliorate cognitive deficits in a mouse model of Down syndrome [70–72]. In our data, where C57BL/6J mice were uniformly exposed to the same dose of PTZ, ictal and interictal changes in activity and sheltering patterns were aligned with the occurrence of a convulsion (Figs 2G and S4C), suggesting that more pervasive changes in behavior varied with regards to induced seizure severity rather than GABA-A receptor antagonism *per se*. Home-cage experiments that incorporate distinct acute seizure models (electrical stimulation, kainic acid, etc.) or established models of acquired epilepsy (pilocarpine or kainic acid status epilepticus) may offer additional insights that clarify the generalizability of these findings with PTZ.

## Conclusions

As we continue to expand our knowledge of epilepsy genetics, innovate mouse models that recapitulate those genetic lesions, and apply novel anatomically targeted methods to interrogate network dysfunction in epilepsy, we must continue to refine our assessment of seizure severity and interictal behavior in preclinical models. Our data illustrates the utility of continuous long-term home-cage monitoring in assessing these important components of epilepsy disability in mice. Future studies that incorporate home-cage monitoring may help dissect the various determinants of spontaneous interictal behavior, including factors such as type of seizure onset (focal Vs generalized), spontaneous seizure or spike frequency/recency, and the pleiotropic role of an underlying genetic etiology (if present). Finally, this technology may also help discern why certain anticonvulsants, regardless of epilepsy type or seizure frequency, are themselves associated with high rates of adverse behavioral side effects [73, 74].

## Supporting information

S1 Fig Validating behaviorally defined “sleep” and group randomization. **(A)** A separate group of 8-10week old C57BL/6J mice were acclimated to home cage chambers for two days and then received either an injection of saline (n = 15, 7F) or diazepam (3mg/kg, n = 16, 8F) at ~1655. Diazepam-treated mice displayed significantly greater average total sleep with a prominent increase in sleep bouts between 1700–2300. Consistent with a sedative effect, diazepam also reduced overall distances moved (group x hour, F_17,493_ = 2.1, p<0.01) and increased sheltering (group x hour, F_17,522_ = 1.7, p<0.05). **(B)** After two consecutive days of baseline recording, 32 C57BL/6J mice (from Fig 1) were randomized to receive saline (n = 12) and PTZ (n = 20). Distances (group x hour, F_20,600_ = 1.3, p>0.1), sheltering (group x hour, F_20,600_ = 1.3, p>0.1) and feeding entries (group x hour, F_20,600_ = 1.1, p>0.1) were not significantly different. **(C-E)** Groups were also similar in total distance moved, sucrose preference and total sleep. ***: p<0.01. Mean ± SEM shown.
(TIF)

S2 Fig Injections of saline Vs PTZ (30mg/kg). LEFT (distances), CENTER (sheltering) and RIGHT (feeding entries) for the 2^nd^ through 9^th^ injections with dot plots reflecting the latency to convulsions in each ictal recording. Mean ± SEM shown.
(TIF)

S3 Fig Individual differences in response to PTZ. **(A)** Over the 10-dayprotocol, 9 mice displayed convulsions following any PTZ injections. On their second baseline day (Fig 1), compared with the 11 mice that never convulsed, these mice displayed a trend to accumulate greater distances (group x hour F_20,360_ = 1.4, p = 0.1), altered sheltering patterns (group x hour F_20,360_ = 2.0, p<0.01) without changes in feeding entries (group x hour, F_20,360_ = 0.5, p>0.9). RIGHT: Time budgets for these groups. **(B)** Behavioral response to the FIRST injection of PTZ in mice that never convulsed (n = 11) and those that ever convulsed (n = 7). We exclude two mice that convulsed following the first injection. **(C)** Averaged across all ten days, PTZ injections produced a mean behavioral response that was clearly distinct from saline. *: p<0.05. Mean ± SEM shown.
(TIF)

S4 Fig Interictal behavior during and following 10 injections of PTZ or saline. **(A)** Across 10 “interictal” recordings (1600–1100, corresponding to data in Fig 3B), PTZ- and saline-treated mice displayed similar total licking (group x day, F_9,261_ = 1.1, p>0.1) or sucrose preference (group x day, F_9,261_ = 0.4, p>0.5). **(B)** Across all interictal recordings, those which followed convulsive seizures were associated with more pronounced nocturnal hypoactivity and sheltering behavior compared with nonconvulsive seizures. **(C)** Other behavioral parameters measured (for Fig 4B) were no different between saline and PTZ-treated mice on measures of sheltering (group x hour, F_19,513_ = 0.3, p>0.9), licking (group x hour, F_19,513_ = 0.4, p>0.9) or feeding (group x hour, F_19,513_ = 0.5, p>0.9). Mean ± SEM shown.
(TIF)

S5 Fig Injections of PTZ in C57BL/6J vs DBA/2J. LEFT (distances), CENTER (sheltering) and RIGHT (feeding entries) for the 5^nd^ through 10^th^ injection with dot plots reflecting the latency to convulsions in each ictal recording. BOTTOM: Overall survival curve (includes ictal recordings depicted in Fig 5). Mean ± SEM shown.
(TIF)

S1 Movie An example of early immobility outside the shelter. In this video (which captures behavior ~7.5 minutes post-injection), the mouse on the top right chamber displays a bout of prolonged immobility following PTZ. Saline-treated mice (all others in this video) are seen within their shelters either grooming or resting.
(MP4)

S2 Movie Integrated visualization for the detection of convulsive seizures. In this video, aerial footage from home-cage chambers are shown on the left with simultaneous measures of “% mobility” on the right (defined as the percentage of changed in pixels of an object between individual samples). In this video, the mouse in the top right experiences a convulsive seizure with hindlimb extension marked by a polyphasic transient change in mobility.
(MP4)

S3 Movie A representative example of a convulsive seizure outside the shelter (top right chamber). (MP4)

S4 Movie A representative example of a convulsive seizure within the shelter (bottom right chamber). (MP4)
