# Supplementary figures and images for: Home-cage monitoring ascertains signatures of ictal and interictal behavior in mouse models of generalized seizures
Source: PLoS One. 2019 Nov 7;14(11):e0224856. doi: 10.1371/journal.pone.0224856 (PMC6837443; doi:10.1371/journal.pone.0224856)

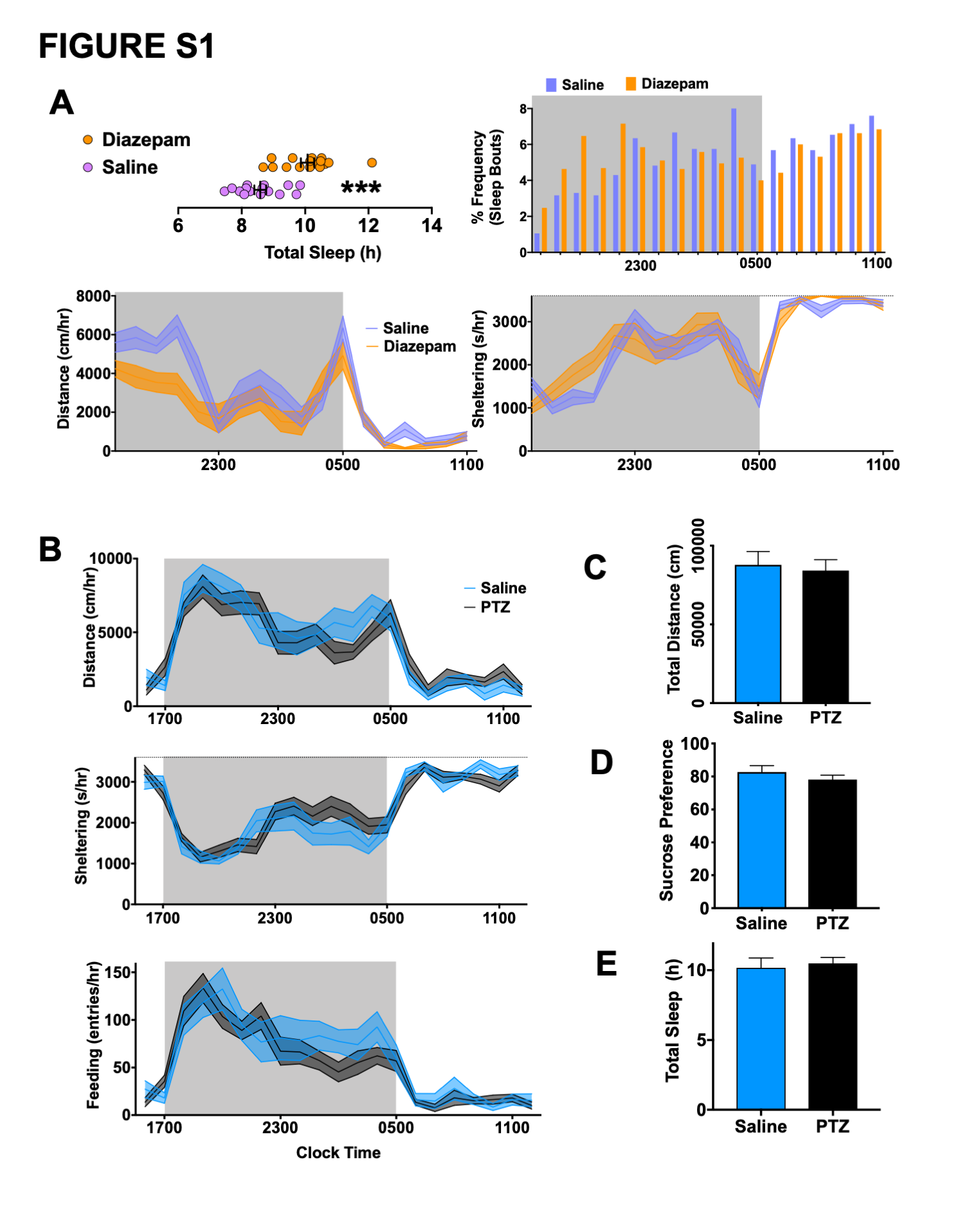

Supplement: S1 Fig — (A) A separate group of 8-10week old C57BL/6J mice were acclimated to home cage chambers for two days and then received either an injection of saline (n = 15, 7F) or diazepam (3mg/kg, n = 16, 8F) at ~1655. Diazepam-treated mice displayed significantly greater average total sleep with a prominent increase in sleep bouts between 1700–2300. Consistent with a sedative effect, diazepam also reduced overall distances moved (group x hour, F17,493 = 2.1, p<0.01) and increased sheltering (group x hour, F17,522 = 1.7, p<0.05). (B) After two consecutive days of baseline recording, 32 C57BL/6J mice (from Fig 1) were randomized to receive saline (n = 12) and PTZ (n = 20). Distances (group x hour, F20,600 = 1.3, p>0.1), sheltering (group x hour, F20,600 = 1.3, p>0.1) and feeding entries (group x hour, F20,600 = 1.1, p>0.1) were not significantly different. (C-E) Groups were also similar in total distance moved, sucrose preference and total sleep. ***: p<0.01. Mean ± SEM shown. (TIF) [file pone.0224856.s001.tif]

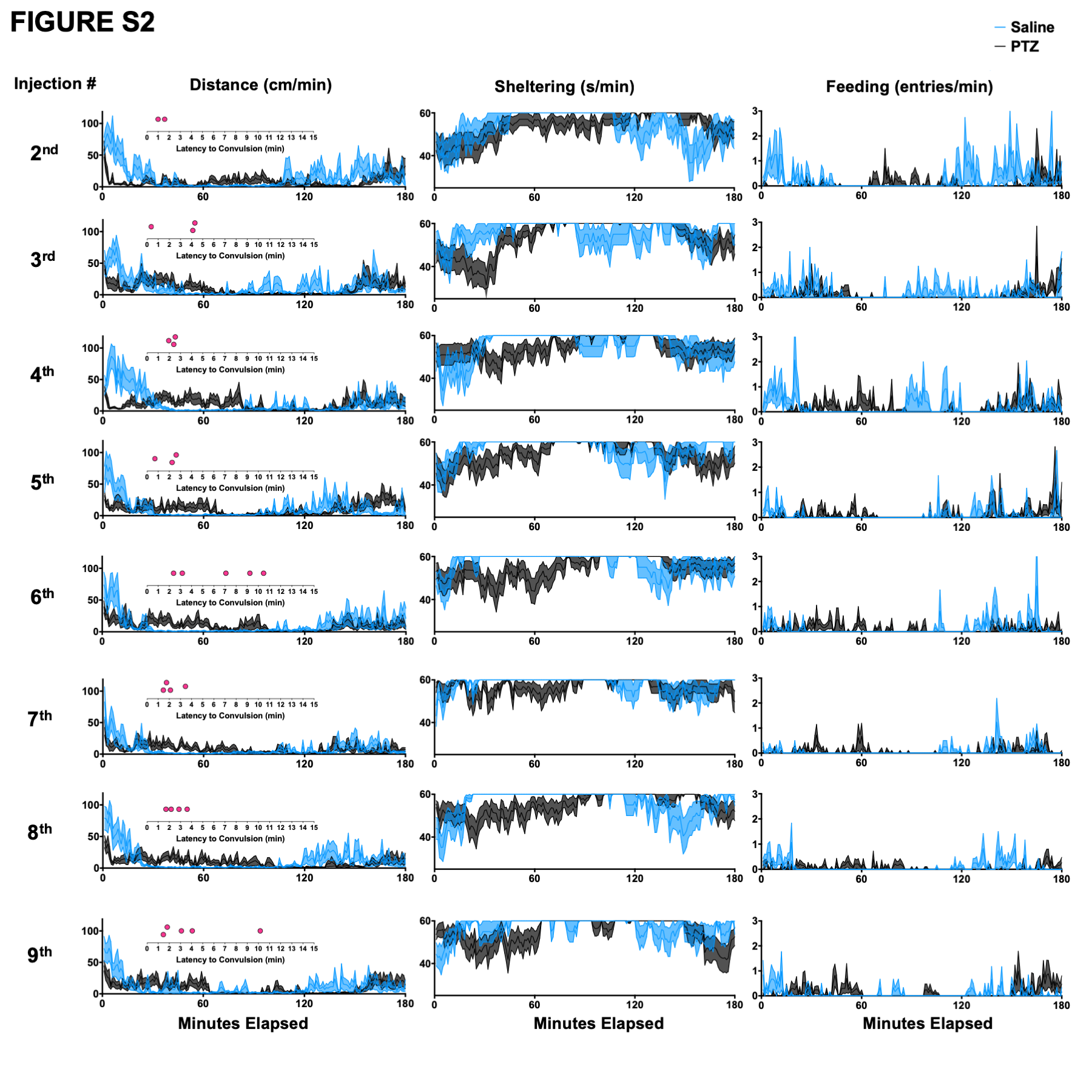

Supplement: S2 Fig — LEFT (distances), CENTER (sheltering) and RIGHT (feeding entries) for the 2nd through 9th injections with dot plots reflecting the latency to convulsions in each ictal recording. Mean ± SEM shown. (TIF) [file pone.0224856.s002.tif]

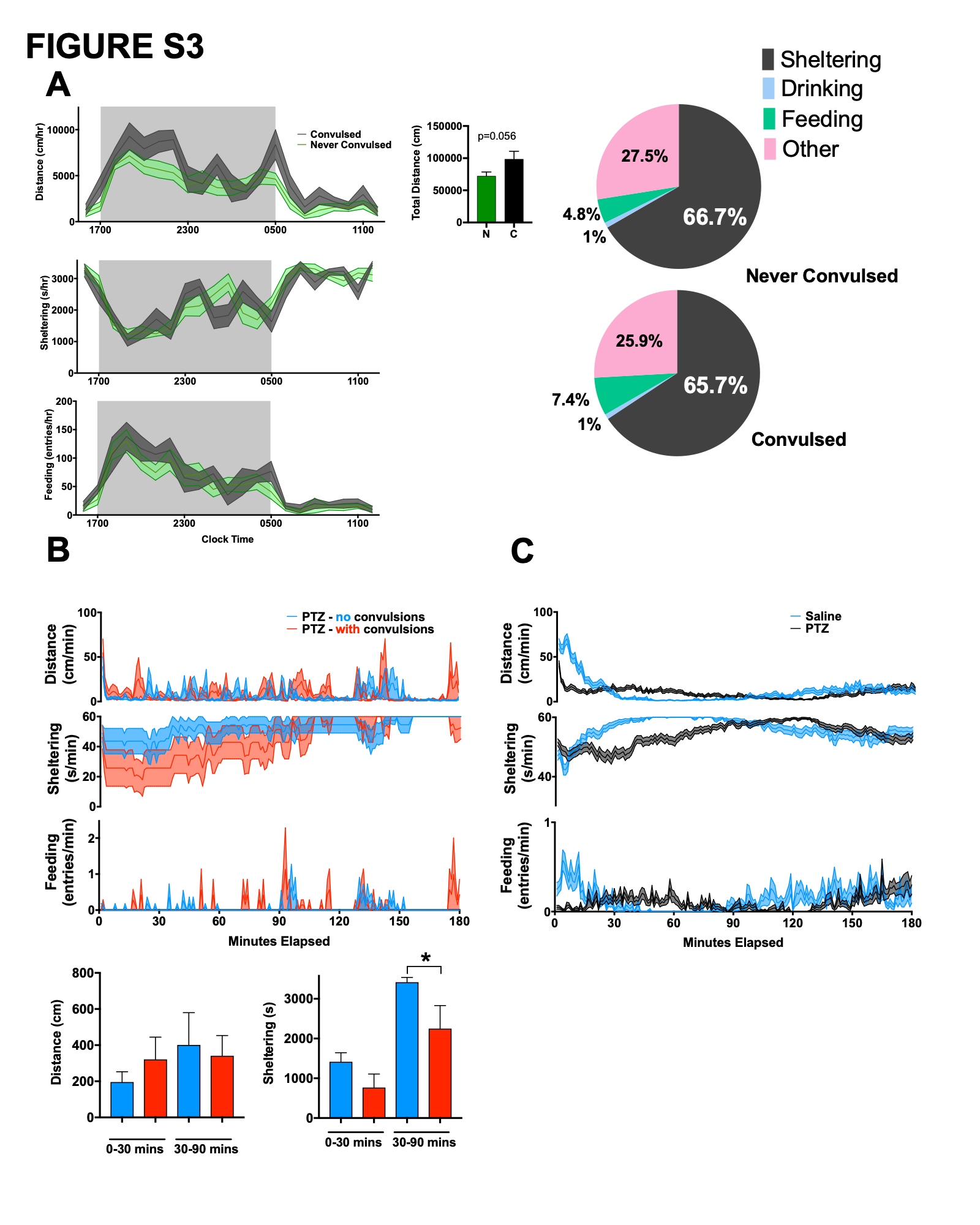

Supplement: S3 Fig — (A) Over the 10-dayprotocol, 9 mice displayed convulsions following any PTZ injections. On their second baseline day (Fig 1), compared with the 11 mice that never convulsed, these mice displayed a trend to accumulate greater distances (group x hour F20,360 = 1.4, p = 0.1), altered sheltering patterns (group x hour F20,360 = 2.0, p<0.01) without changes in feeding entries (group x hour, F20,360 = 0.5, p>0.9). RIGHT: Time budgets for these groups. (B) Behavioral response to the FIRST injection of PTZ in mice that never convulsed (n = 11) and those that ever convulsed (n = 7). We exclude two mice that convulsed following the first injection. (C) Averaged across all ten days, PTZ injections produced a mean behavioral response that was clearly distinct from saline. *: p<0.05. Mean ± SEM shown. (TIF) [file pone.0224856.s003.tif]

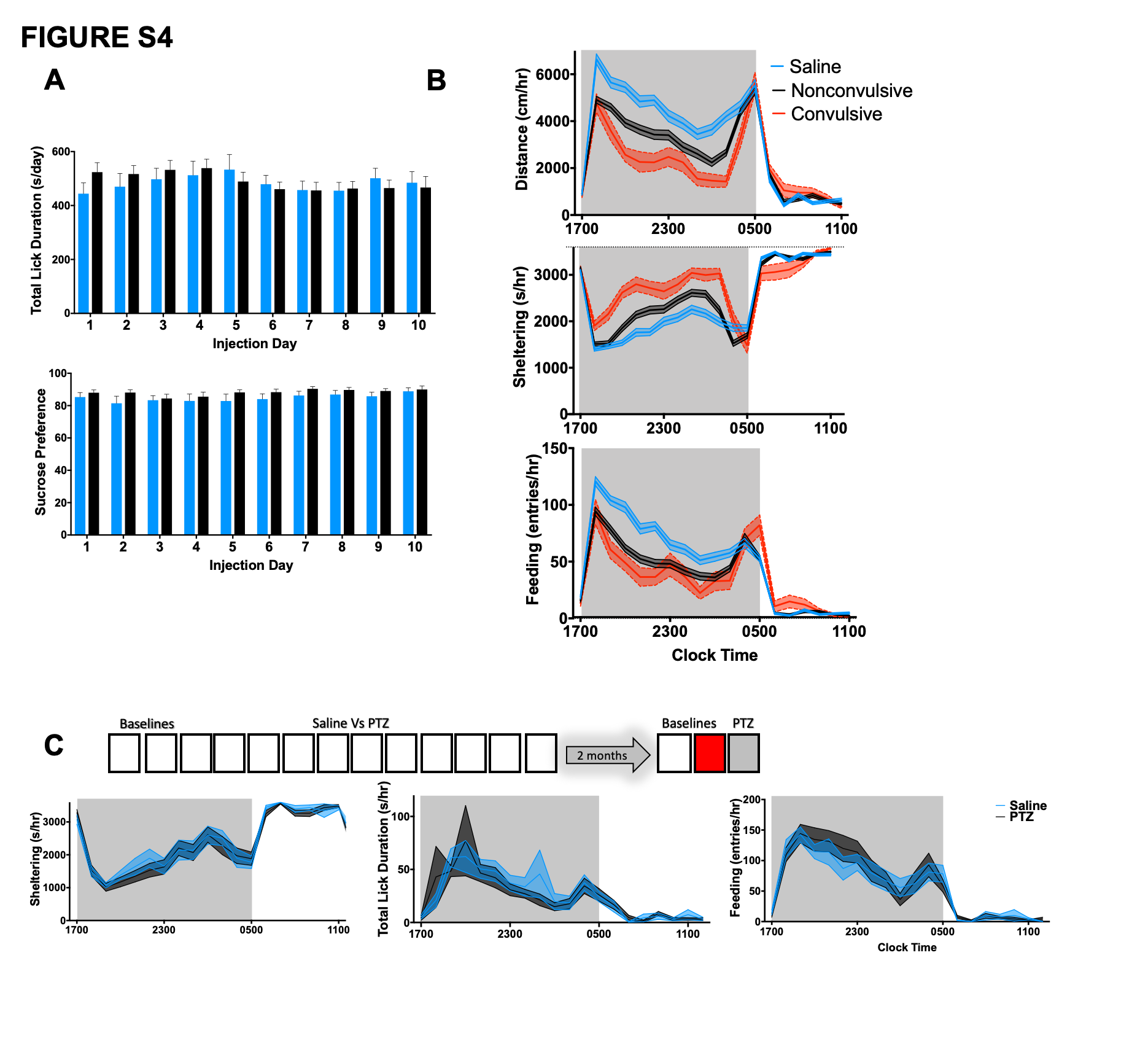

Supplement: S4 Fig — (A) Across 10 “interictal” recordings (1600–1100, corresponding to data in Fig 3B), PTZ- and saline-treated mice displayed similar total licking (group x day, F9,261 = 1.1, p>0.1) or sucrose preference (group x day, F9,261 = 0.4, p>0.5). (B) Across all interictal recordings, those which followed convulsive seizures were associated with more pronounced nocturnal hypoactivity and sheltering behavior compared with nonconvulsive seizures. (C) Other behavioral parameters measured (for Fig 4B) were no different between saline and PTZ-treated mice on measures of sheltering (group x hour, F19,513 = 0.3, p>0.9), licking (group x hour, F19,513 = 0.4, p>0.9) or feeding (group x hour, F19,513 = 0.5, p>0.9). Mean ± SEM shown. (TIF) [file pone.0224856.s004.tif]

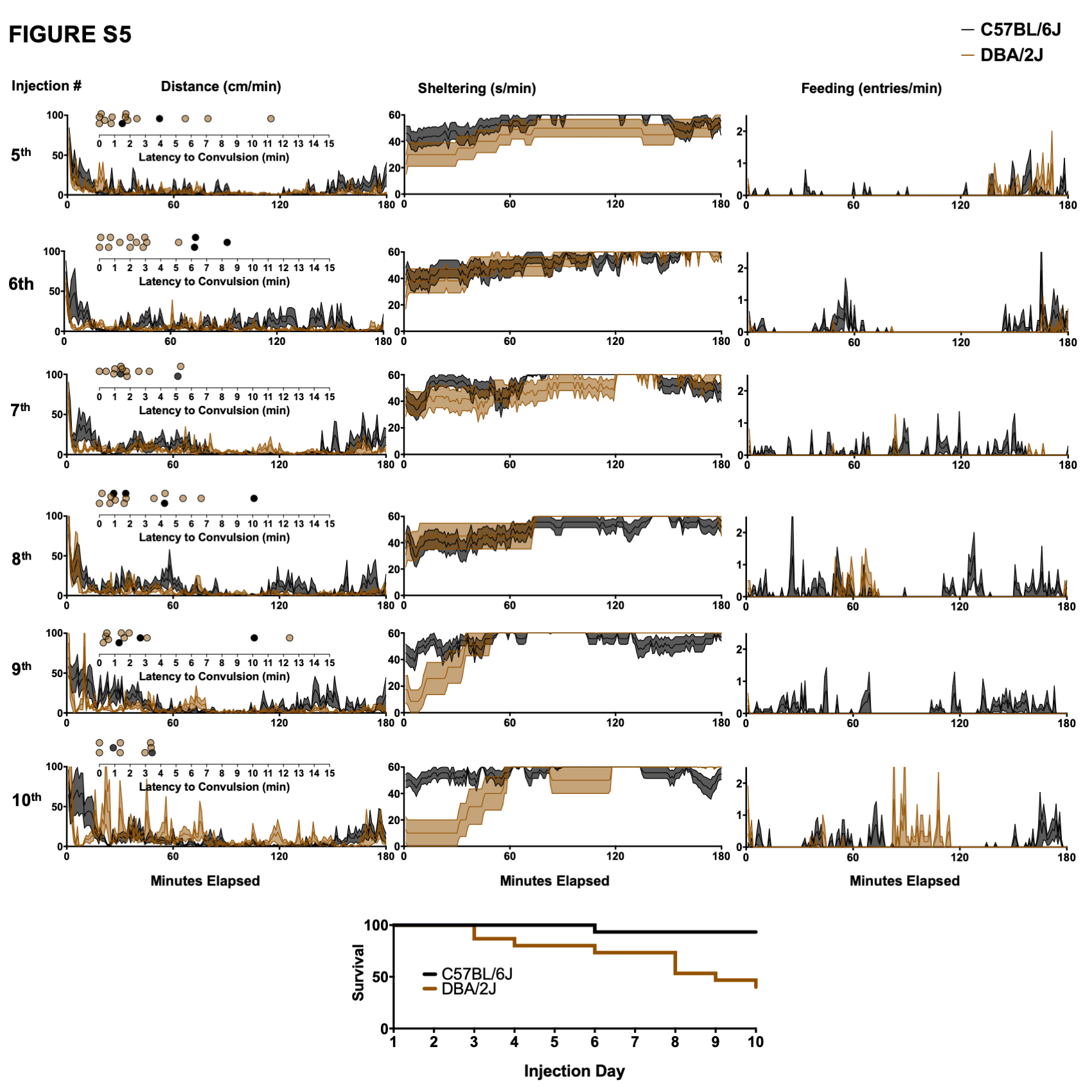

Supplement: S5 Fig — LEFT (distances), CENTER (sheltering) and RIGHT (feeding entries) for the 5nd through 10th injection with dot plots reflecting the latency to convulsions in each ictal recording. BOTTOM: Overall survival curve (includes ictal recordings depicted in Fig 5). Mean ± SEM shown. (TIF) [file pone.0224856.s005.tif]
